# Environmental Triggers of Autoreactive Responses: Induction of Antiphospholipid Antibody Formation

**DOI:** 10.3389/fimmu.2019.01609

**Published:** 2019-07-10

**Authors:** Anush Martirosyan, Rustam Aminov, Gayane Manukyan

**Affiliations:** ^1^Laboratory of Molecular and Cellular Immunology, Institute of Molecular Biology, Yerevan, Armenia; ^2^Russian-Armenian (Slavonic) University, Yerevan, Armenia; ^3^School of Medicine, Medical Sciences and Nutrition, University of Aberdeen, Aberdeen, United Kingdom

**Keywords:** antiphospholipid antibodies, antiphospholipid syndrome, bacteria, viruses, vaccination, drugs

## Abstract

Antiphospholipid antibodies (aPLs) comprise a diverse family of autoantibodies targeted against proteins with the affinity toward negatively charged phospholipids or protein-phospholipid complexes. Their clinical significance, including prothrombotic potential of anti-cardiolipin antibodies (aCLs), anti-β2-glycoprotein I antibodies (aβ2-GPIs), and lupus anti-coagulant (LA), is well-established. However, the ontogeny of these pathogenic aPLs remains less clear. While transient appearance of aPLs could be induced by various environmental factors, in genetically predisposed individuals these factors may eventually lead to the development of the antiphospholipid syndrome (APS). Since the first description of APS, it has been found that a wide variety of microbial and viral agents influence aPLs production and contribute to clinical manifestations of APS. Many theories attempted to explain the pathogenic potential of different environmental factors as well as a phenomenon termed molecular mimicry between β2-GPI molecule and infection-relevant structures. In this review, we summarize and critically assess the pathogenic and non-pathogenic formation of aPLs and its contribution to the development of APS.

## Introduction

Antiphospholipid antibodies (aPL) comprise a diverse family of heterogeneous autoantibodies targeting proteins with the affinity toward negatively charged phospholipids or protein-phospholipid complexes. aPLs are increasingly being recognized as causative factors of antiphopholipid syndrome (APS) manifestations. Syndrome is defined by persistent aPL positivity accompanied by thromboembolic events and/or obstetrical complications (1).

While a broad spectrum of aPL exists, the presence of anti-β2 glycoprotein I (anti-β2GPI) antibodies, anticardiolipin (aCL), and lupus anticoagulant (LA) is accepted as independent risk factors for the episodes of vascular thrombosis and pregnancy loss in APS (2, 3). aPL are responsible for the alterations in the mechanisms of coagulation and fibrinolysis, leading to a proinflammatory, or hypercoagulable state. Beyond the heterogeneity of different aPL, it is widely accepted that they represent a bulk of natural antibodies (mainly IgG and IgM classes) with low affinity, generated for the most part by B1 cells (4). Pathogenic role of aPL was shown in animal studies (5, 6). In mice, the passive transfer of aPL produced features resembling human APS, that is, fetal loss and thrombotic events (7). Another evidence for the pathogenicity of autoantibodies was obtained in studies proving a substantial clinical improvement after the removal of autoantibodies by plasma exchange or plasmapheresis (8). Mechanistically, we demonstrated the restoration of monocyte transcriptional activity in patients with APS after plasmapheresis (9).

The presence of aPL is not necessarily associated with the primary APS; they could be detected in a variety of clinical conditions, including different autoimmune diseases, infections, neoplasms, after vaccination or drug use, etc., and could also be transiently expressed in healthy individuals (3). In autoimmune disorders, aPL are mainly cofactor dependent and require the presence of phospholipid-binding proteins, such as β2GPI and prothrombin, which are their main targets, while aPL generated after infections are largely cofactor independent (10).

Based on the currently available clinical and experimental evidence, it is likely that in predisposed individuals, different environmental triggers may eventually lead to the production of aPL, which then can lead to the development of APS. Several theories tried to explain the pathogenic potential of different environmental factors as well as a phenomenon termed molecular mimicry between β2GPI molecule and infection-relevant structures. This review summarizes current findings on the origin and mechanisms of production of APS-associated antibodies and compiles the recent advances toward understanding the functional relevance of aPL to the development of APS.

## Viruses

Among a wide range of infectious agents, which may contribute to the increase of aPL, human immunodeficiency virus (HIV) and hepatitis C virus (HCV) are among the most frequently reported. HIV infection, association of which with aCL antibodies has been first reported more than 30 years ago (11), currently remains the most frequent infectious cause for aPL production in adult and pediatric patients. A considerable diversity of aPL can be detected in HIV-infected individuals, including aCL, anti-β2GPI, LA, aPT, aPS, aPI, and aPC (12, 13). The aCL antibodies during HIV infection could be of both pathogenic type (β2GPI cofactor dependent) and post-infectious type (non-β2GPI dependent). The estimated incidence of IgG aCL among HIV-positive individuals is in the range of 7–94% (mean 46.5%) compared to 1.98% in control subjects (13). IgM aCL are less common and their clinical significance remains controversial (14). Both positive (15) and negative (14) associations between aCL and thromboembolic phenomena in patients were reported, although aPL-related clinical features are generally uncommon in HIV patients (16). The latter may be explained by the ability of β2GPI to inhibit the interaction between HIV-specific IgG aPL antibodies and CL, which was demonstrated in the competition study designed to delineate the true autoimmune and non-pathogenic aPL (17). Further assessment of the binding specificity and avidity of aPL antibodies in HIV showed low antibody avidity (18). Except for a low incidence of anti-β2GPI, the prevalence of aCL, aPS, aPI, and aPC antibodies in HIV-1 infection was comparable to that found in APS, indicating the tendency to recognize various phospholipids other than β2GPI (18). It is likely that the relatively low levels of anti-β2GPI and cofactor-independent nature of HIV-specific aPL, compared to the autoimmune one, accounts for the rare incidence of APS among HIV-positive individuals. The frequency and complications of LA may vary considerably among HIV-infected patients. Reported incidences are in the range of 0–72% (19), but the pathogenic association with thrombosis is somewhat exceptional (15).

Similarly to HIV, aCL antibodies are the most common type of aPL in HCV infection. A high prevalence of aCL in HCV patients compared to healthy controls has been reported (18.6% vs. 1.78%) (13). aCL from these patients showed no β2GPI dependency, and association with the thromboembolic events was weak (15). An earlier study demonstrated that aCL due to HCV exhibit features of natural polyspecific autoantibodies, suggesting their non-pathogenic nature (20). High incidence of LA presumably predispose the occurrence of thrombotic complications in a small subset of HCV-positive APS patients, since in an unselected cohort of HCV-infected individuals the presence of LA is a rare event (21). Albeit other hepatitis viruses were less intensively studied in term of aPL, occurrence of aCL in both HBV and HDV infection have been suggested to interfere with thrombotic incidence in patients with hepatitis-related hepatocellular carcinoma (22).

High frequency of HIV- and HCV-induced aPL in terms of a possible association with APS is highly attractive but also controversial. Analyses of the spectrum of clinical features related to APS (peripheral thrombosis, valvular heart disease, nephropathy, etc.) in patients with chronic HIV/HCV infections suggested that while in individual cases these infections may lead to the development of a true APS, in most cases the presence of aPL and relevant clinical manifestation might merely represent an epiphenomenon secondary to the chronic immune stimulation by the virus (21). In several documented cases, thrombotic episodes in HIV, or HCV-infected patients were linked to extrinsic causes (antiretroviral treatment and opportunistic infections), suggesting a limited role of aPL in these events (12). Analogously, HIV-associated vascular damage, endothelial dysfunction and pulmonary embolism may well mimic clinical presentation of APS. In the case of HCV, immunological markers of chronic infection such as ANA, cryoglobulins, hypocomplementemia, and rheumatoid factor may potentially contribute to the development of symptomatic features resembling APS.

In the light of remarkable similarities in the clinical manifestations of patients with parvovirus B19 infection, autoimmune systemic lupus erythematosus (SLE), and/or APS such as thrombosis, hemolytic anemia, spontaneous abortion, livedo reticularis, and arthritis, B19 infection is considered as an initial trigger for autoimmune processes (23). Development of pathogenic aPL has been often reported as being associated with this infection. In particular, over a quarter of children with rheumatic diseases were found to be positive for aPL and also had B19 parvovirus infection, presently or in the past (24). The most notable characteristic of aPL, detected in patients infected with parvovirus B19, is the enhanced antigen-binding ability in the presence of β2GPI as a cofactor (25). Antibodies present in the serum of B19-infected patients are predominantly of the IgG isotype, they are specific toward the negatively charged phospholipids, aCL and phosphatidyl serine (aPS) and, on rare occasions, to the neutral phospholipid phosphatidyl ethanolamine (aPE). Based on these observations, it has been suggested that the nature of B19-induced aPL is markedly different from the antibodies generated in response to other viral infections. Their binding ability, isotype distribution, and cofactor dependence resemble those present in patients with autoimmune diseases, pointing to the potential role of B19 in triggering autoimmune events (25). To our knowledge, there have been at least two reported cases of thrombotic events associated with parvovirus B19 infection: a male patient who developed splenic infarction and a female patient with multiple pulmonary emboli (26, 27). Both cases were characterized by the transient presence of aPL antibodies of IgM or IgG isotypes. Mechanisms responsible for the aPL production during B19 infection are: cross-reactivity between anti-B19 IgG antibodies with multiple human autoantigens (cardiolipin, collagen II, keratin, angiotensin II type 1 receptor, and platelet membrane glycoprotein IIb/IIIa), presentation of apoptosis-associated self-antigens by infected cells (23), and phospholipase-A2-like activity observed in the VP1 unique region of the structural protein VP1 (24).

Viral agents that are less frequently reported as provoking a transient or permanent rise of aPL include the human herpesviruses such as cytomegalovirus (CMV), varicella-zoster virus (VZV), and Epstein-Barr virus (EBV) (28). They are common viral pathogens with the estimated seroprevalence among adult population >90% (29). These three viruses have been implicated in the presence of aPL and aPL-related clinical manifestations. An intriguing association between infection and aPL was revealed in pregnant women who underwent routine prenatal screening for toxoplasmosis, rubella, cytomegalovirus, and herpes simplex virus (TORCH panel). Substantial proportion of healthy pregnant women (52.2%) with false-positive TORCH appeared to be aPL positive (30). Another study has summarized the findings on thrombosis associated with CMV infection: among 97 patients who developed CMV-induced thrombosis 14 had transient or permanent APS (31). aPL-independent mechanisms responsible for CMV-triggered procoaguable state include activation of coagulation factors and platelets, adhesion of leukocytes as a sequel to the direct vascular endothelial damage by the virus (32), generation of thrombin or factor VIII, and/or down-regulation of physiological anticoagulant mechanisms (33). Another cross-sectional study, with 95 children enrolled, showed a transient increase of LA and aCL in 43 children following varicella infection (34). Moreover, a direct pathogenic role of VZV-induced transient increase of IgM/IgG aCL, IgM anti-b2GPI, or LA in the development of hypercoaguable state was suggested for a number of pediatric and adult cases with thrombotic or vascular complications (35–37). Exposure to EBV was associated with a lupus phenotype and promoted autoimmune processes (38). The main type of aPL detected in EBV infection was aCL IgG (39). In favor of pathogenic nature of aPL is the case described in the report by Delbos et al., who described CMV-induced thrombosis in a previously healthy woman with a pulmonary embolism in the presence of aPL (40).

Adenovirus infection is also associated with a significant increase of aCL. aCL-specific IgG and IgM classes were found in a half of children with respiratory disease caused by adenoviral infection (41). Transitory increase of LA, aCL, and antiphosphatidylserine-dependent antithrombin antibodies (aPS/PT) was observed in a 9-year-old girl following acute gastroenteritis (due to adenovirus) and pneumonia (due *Mycoplasma pneumoniae*) (42). Herpes simplex virus, dengue virus, human herpes virus 6, rubella, measles, chicken pox, and mumps infections are usually accompanied by a relatively low level of autoantibodies, which are often declined after the elimination of infection and are not associated with thrombosis incidences (28).

Initial encounter between a virus and a target cell occurs at the plasma membrane interface. Negatively charged phospholipids, along with low-density lipoprotein receptors and gangliosides, have been shown to play a role in the entry of numerous viruses (43). Most enveloped viruses, including retroviruses, acquire their lipid envelope by budding through the cellular membrane. For example, HIV uses phosphatidylserine (PS) as a cofactor for infection and acquires this phospholipid from lipid rafts during budding (44). A comprehensive survey of total lipid composition of retroviral envelopes revealed a considerable resemblance with that of the plasma membrane, unless they were highly enriched in specific negatively charged phosphoinositides, PIP and PIP2, which are phosphorylated derivatives of phosphatidylinositol (45). Additionally, viruses use the host lipids in the later stages of infection to promote intracellular trafficking, replication, viral assembly, and egress. RNA and DNA viruses that replicate in the cytosol tend to entail membranes of specific cellular compartments (e.g., endoplasmic reticulum, mitochondria, endosomes, lysosomes, or Golgi bodies) (46). Despite that the mitochondrial disturbances are prevalent among HIV-infected individuals, interaction of the virus with host mitochondrial CL has not been tested yet. However, demonstration of *in vitro* CL cross-reactivity with HIV–specific neutralizing antibodies (47) has inspired researchers to analyze the correlation between the occurrence of HIV-specific aCL and viral parameters. It was shown that aCL, detected in HIV patients, are strongly and independently associated with the level of virus replication, regardless of the disease stage (48). In another case report, initially high titers of aCL and anti-β2GPI in a HIV-positive man were shown to progressively decrease, in parallel with a viral load (49).

It seems that entangled and interactive relationships between the viral infection and the host immune system mediate diversity of aPL responses and related manifestations. Despite the mounting evidence supporting the contribution of infection agents to production of autoantibodies, aPL-related complications in the context of viral infection are rare. Thus, the development of true APS is exceptional, excluding the cases when autoimmunity is present prior infection. In this context, the assessment of β2GPI dependence could be helpful in differentiating the pathogenic autoimmune aPL from post-infectious one.

## Bacteria

It is a widely accepted view that pathogenesis of many autoimmune diseases is largely driven by inappropriate or inadequate immune responses toward bacterial agents (50, 51). Similarly, a number of Gram-positive and Gram-negative bacteria are recognized as being linked to aPL production. Despite this, transitory increase of bacteria-induced aPL autoantibodies was only occasionally associated with thrombotic events (52).

*M. pneumoniae* and *Streptococcus* spp. infections, which are among the most prevalent bacterial infections in children and young adults, were linked to the occurrence of aPL. A significant increase in aCLof IgM and IgG classes was found in patients with *M. pneumoniae*, especially in those with severe infection or with cold hemagglutinins (53). Almost a half of patients with poststreptococcal glomerulonephritis were positive for aCL, with a long-term persistence of the antibodies in the majority of patients, but with no thromboembolic events (54). A case of childhood-onset autoimmune APS and several pediatric cases of aPL-related thromboembolic complications at various anatomical sites were all attributed to the pulmonary *M. pneumonia* infection (55–57). Two independent studies reported splenic infarctions secondary to aPL positivity due to *M. pneumoniae* or *Staphylococcus* spp. infection (58, 59).

Another evidence for aPL-mediated thrombosis as a consequence of microbial infection has been recently described in Lemierre syndrome (60). This a rare and potentially life-threatening condition characterized by thrombophlebitis of the internal jugular vein and pulmonary embolism (61). The syndrome is classically associated with an anaerobic bacterium *Fusobacterium necrophorum*, although a variety of other bacteria such as streptococci, staphylococci, and enterococci may be also responsible for the disease (61).

Examination of serum samples from leprosy patients demonstrated the presence of LA and aPT, with the prevalence of anti-β2GPI (62, 63), as well as the heterogeneity of aCL in regards to co-factor dependency (64). Some authors have suggested similarities between the leprosy-mediatedaPL with those found in patients with autoimmune diseases (65). Nevertheless, leprosy-specific aPL are infrequently associated with the thrombotic features. Several exceptional cases of microvascular thromboses, related to β2GPI-dependent aCL, were documented in patients who developed a rare Lucio's phenomenon on a background of diffuse lepromatous leprosy (66).

Although somewhat controversial, syphilis infection has also been associated with the production of aPL. The reported prevalence of syphilis-related aCL is wide-ranging, varying from 8 to 67%, which may be attributed to their cross-reactivity with treponemal cardiolipins (13). Nevertheless, the presence of anti-β2GPIand LA and thrombotic episodes are uncommon in this infection (13).

The pathogenic potential of aPL found in patients infected by *Mycobacterium tuberculosis*is contentious. Poly-reactive B1 cells were shown as the main source of non-specific anti-phospholipid IgM antibody produced in response to *M. tuberculosis* lipids which suggests non-pathogenic nature of these aPL (67). In support of this assertion no association of aPL levels with thrombotic events was reported. The increased levels of aCL-specific IgM and IgG isotypes were found in several population-based cohort studies (68–70). Elkayam et al. (71) were first to reveal the elevated level of anti-β2GPI in a substantial proportion of tuberculosis patients, which is declined following TB therapy (71). Similarly, the normalization of IgM class of aCL, antiphosphatidyl inositol (aPI), antiphosphatidyl ethanolamine (aPE), antiphosphatidyl choline (aPTC), and antisphingolipid (aSL) antibodies in the sera of tuberculosis patients following TB therapy was reported (72).

Presently it is widely accepted that infection by *Helicobacter pylori* is the most common cause of chronic gastritis (73). Both the pathogenic and protective roles for this infection in different autoimmune diseases were suggested (74). A first large-cohort study has been performed to estimate the prevalence of anti-*H. pylori* (anti-HP) antibodies in 1,290 patients diagnosed with 14 different autoimmune diseases, including 157 primary and secondary APS patients, and 385 matching healthy controls (75). All subjects were screened for the presence aPL. The study uncovered the highest frequency of anti-HP IgG and significantly elevated levels of aCL IgM among the patients with primary APS (75). Another study demonstrated a causal relationship between *H. pylori* infection and aCL of all isotypes IgA/IgM/IgG in 84 children, with a decline of autoantibody titers following eradication of *H. pylori* (76).

## Yeasts and Fungi

The role of yeasts and fungi in APS has not gained too much attention and thus the cases of fungal-induced aPL are very rare in the literature (28). Recent findings have raised wariness regarding a pathogenic potential of food products prepared with the use of *Saccharomyces cerevisiae*, known as Brewer's and Baker's yeast (77). Anti-*S. cerevisiae* antibodies (ASCAs), directed against the phosphopeptidomannan, have been demonstrated to be prevalent among aPL-positive autoimmune patients, including those with Crohn's disease (78), SLE (79), RA (80), and APS (81). The presence of cross-reactive epitopes on β2GPI and *S. cerevisiae* (77, 81) and the fact that ASCAs tend to appear years before the fully developed autoimmune conditions (82) point to a possible pathogenic significance of ASCAs in APS.

## Commensal Microbiota

Besides directly affecting the host, diet may exert indirect influence via modulating the composition and function of the gut microbial communities. Ingestion of probiotics, for instance, is considered to be beneficial for modulation and/or restoration of the gut microbiota, and it is capable of altering the course of autoimmune diseases and APS. For example, consumption of probiotic fermented milk products in β2GPI-immunized Balb/c mice resulted in a decline of serum anti-β2GPI titers and in the shift from Th2 type immune response toward Th1 (83). Based on above-mentioned observations, a role for commensal bacteria was hypothesized (84). Indeed, a report by Aguiar and coworkers has shown a specific change in the gut microbial composition in APS patients. Particularly, a decrease of bacteria belonging to the genus *Bilophila* and overgrowth of bacteria of the *Slackia* genus were shown (85). Moreover, anti-domain I (DI) β2GPI IgG positivity in APS patients correlated significantly with the enrichment by *Slackia* spp. and by the lower abundance of butyrate-producing *Butyricimonas* (85). Reduction in microbe-derived butyrate, which has an anti-inflammatory and immunomodulatory activity (86), may enable a stronger T-cell proliferative response against β2GPI. Antigen-specific response toward gut microbiota was proven by the administration of broad-spectrum antibiotics in APS-prone (NZWxBXSB)F1 mice, which diminished the proliferation of β2GPI-reactive T cells but not of anti-CD3 (87). These microbiota-depleted animals showed a higher survival rate due to the prevention of thrombotic events and suppression of serum anti-β2GPI IgG titers. In further developments, a specific Gram-positive candidate bacterium, involved in immune cross-reactivity, has been identified based on *in silico* analysis. Common colonic butyrate-producing *Roseburia intestinalis* shared a high degree of sequence homology with both the major B and T cell epitopes to β2GPI (84). The evidences of the involvement of commensal microbiota in aPL production are sporadic and mainly published as preliminary results which does not allow us to fully estimate their contribution. Need to reiterate here though that under the normal conditions the epitopes of commensal bacteria are not usually exposed to the adaptive arm of the immune system. Response to the epitopes of commensal microbiota may appear only if the epithelial barrier function is compromised thus allowing elevated translocation of bacteria and the exposure of epitomes that are usually not encountered under the normal circumstances.

Commensal bacteria may act in several ways to modulate immune reactions. Initiation of an autoimmune reaction by gut commensals is likely to engage the mechanisms similar to that of pathogenic microbiota. Among the different potential scenarios, cross-reactivity triggered by commensal cardiolipin seems to be most intriguing. Cardiolipin is an ancient phospholipid universally found in energy transducing membranes of both prokaryotes and eukaryotes, albeit at different locations (88). In eukaryotes CL is found almost exclusively in mitochondria, while bacterial CL is found in the plasma membranes (89), which reflects the endosymbiotic origin of mitochondria. Given the fact that human CL is normally sequestered from the immune system but the aCL antibodies are broadly prevalent among infected/autoimmune patients, bacterial CL could be considered as a potential damage-associated molecular pattern (DAMP). Thus, it is reasonable to assume that CL-containing bacteria, among which Gram-positives are especially enriched with CL (90), can act as a source of persistent cross-reactive antigens in a genetically susceptible host with a compromised self-tolerance. Spectroscopic analysis has demonstrated a conformational change in β2GPI upon binding to CL, resulting in generation of highly immunogenic epitopes within β2GPI capable of mounting an autoimmune response (91). Therefore, it could be assumed that the initial pathology, which exposes the normally sequestered endogenous and bacterial CLs, may then lead to the recognition by the DAMP receptors and generation of different aPL subpopulations in respect to cofactor dependency.

## Vaccines

Increased medical and public interest toward the safety of vaccination has been heightened following several reports of possible post-vaccination complications involving autoimmune reactions. Causal relationship between several vaccines and autoimmune responses has been reported in numerous studies, among which influenza vaccination is the most frequently reported in the context of aPL generation.

In two pediatric case reports a transient appearance of aPL following inactivated influenza vaccination was demonstrated. A 5-year-old boy and 5-year-old girl were both diagnosed with Henoch-Schönlein purpura (HSP), with the presence of aPL antibodies (92, 93). The involvement of bacterial and viral infections was excluded based on laboratory analyses. The girl presented with a palpable purpuric rash and severe abdominal pain about 12 days after immunization (93). Two months later, her blood was negative for aCL or LA. After 4 months, second immunological examination revealed elevated IgG aCL antibodies. Similar symptoms, accompanied by elevated anti-influenza A/H3N2 antibody, were presented in the boy on the second day after vaccination. Initial palpable purpura, abdominal and ankle pain was successfully improved after a month of oral corticosteroid therapy. Levels of aCL IgG antibody declined within a month, and LA disappeared 2 months after hospital discharge (92).

An association between influenza immunization and systemic autoimmune features was studied in 24 female patients with SLE (94). Six weeks after the vaccine administration, high titers of antibodies reacting with Sm, Sm/RNP, Ro, and La antigens were found, with a reduction in titers after 12 weeks follow-up. Another study of post-vaccination events in SLE patients, with and without former aPL positivity, revealed some mild clinical side-effects and increased levels of anti-β2GPI antibodies of IgG and IgM isotypes after repeated annual influenza vaccination (95). The findings suggested that in clinically stable SLE influenza vaccination may increase the risk of thrombotic manifestations (95). Kinetics of aCL, anti-β2GPI, LA, anti-ENA, and ANA autoantibodies, induced by seasonal influenza vaccination, was analyzed in a large group of healthy adults (96). Increased levels of autoantibodies were found in 15% of participants 1 month after the immunization (96). Six months later, increased levels of autoantibodies remained in 13% of participants suggesting potential long-term effects of influenza vaccination including the risk of thrombotic manifestations.

Although several reports suggested induction of aPL following seasonal and pandemic influenza vaccination, examination of the literature in this field revealed conflicting reports. Vista et al. (97) investigated a large cohort (*n* = 101) of SLE patients and matching controls (*n* = 101) at baseline and 2, 6, and 12 weeks after receiving seasonal influenza vaccination. The study showed a post-vaccination increase of aCL but without the increased onset of β2GPI (97). Another study, which employed a large panel of autoantibodies for screening, investigated 45 primary APS patients and 33 healthy controls after A/H1N1 non-adjuvant vaccine administration. In contrary to the previous study, they failed to find short- or long-term sequelae involving a significant increase of aPL-related antibodies and thrombosis, suggesting that influenza vaccine, at least without adjuvant, is safe (98).

Several case reports raised concerns over a possible association between the vaccine-induced transitory autoimmune responses and the onset of neurological symptoms. An acute confusion state accompanied by severe headache, a middle cerebral artery occlusion, vasculitis, and thrombocytopenia were developed in a 55-years old woman, soon after receiving an influenza vaccine (99). This patient was diagnosed with SLE and secondary APS based on clinical findings and positive for anti-dsDNA, ANA, IgG aCL, and LA. Other two cases of elevated serum IgG anti-phosphatidylcholine antibodies were described in two female pediatric patients with a sudden onset of bilateral optic neuritis due to the administration of trivalent inactivated influenza vaccine (100).

There are only few indications of a possible effect of other types of vaccines on induction of aPL autoantibodies. Fluctuations of aCL, LA, and anti-β2GPI were seen in the minority of participants (*n* = 8/85) after a month following hepatitis B vaccination, with a decline of post-vaccination titers 5 months later (101). Manifestation of definitive APS was proven in a young male as a long-term consequence of diphtheria-tetanus toxoid vaccination (102). The patient experienced thrombotic event accompanied with the stable high LA titer over the next 3 months. Another clinically and serologically proven case of APS was a 13-year-old girl who received a quadrivalent human papillomavirus (HPV) vaccine (103). Acute thrombocytopenia and bleeding were rapidly improved although positive titers of aPL persisted for 6 months.

Among other vaccines, the strongest experimental evidence for post-vaccination development of aPL was revealed for tetanus toxoid (TTd) vaccine. In mouse models, APS manifestation was achieved by immunization of mice with TTd using different adjuvants (104, 105). In humans, an evidence of anti-β2GPI/anti-tetanus toxoid cross-reactive antibody generation after vaccination with TTd in two healthy men was reported (106).

Clearly, vaccines should share molecular patterns with pathogens to elicit protective immune responses. The overall reported frequency of aPL incidences after influenza immunization, however, is higher than after infection (our survey of the published literature). Whether this phenomenon is due to the better studied cohort of vaccinated individuals or to the increased sensitivity to concomitant stimuli such as adjuvants remains unknown. Recently, Shoenfeld and Agmon-Levin suggested to group several autoimmune conditions in a single entity called “ASIA”—Autoimmune (Autoinflammatory) Syndrome Induced by Adjuvants,” which is triggered by external agents such as infections and vaccines (107). It has to be noted, however, that the effects of adjuvants could be significant only in genetically predisposed individuals, whilst less so in general population.

## Drugs

Evidence for a possible association of aPL production and drug administration was first described in 1945 in a patient, who developed a lupus-like syndrome presumably following sulphadiazine therapy (108). Currently a wide range of pharmacological agents used to treat arrhythmia, psychological disorders, hypertension, bacterial infections, epilepsy, or other convulsions, etc. could be implicated in the production of aPL and, in some cases, development of clinically significant APS manifestations (109). Pathogenic potential and clinical manifestations related to drugs-induced aPL are variable and largely dependent on the specific nature of a drug under question. The majority of the drug-induced aPL are usually considered to be fairly benign and disappear after the drug withdrawal (110). aPL induction by procainamide, adalimumab, interferon-α and several other drugs, however, may be linked to the development of thrombosis (111).

Antiarrhythmic agents, including procainamide, and quinidine have been frequently associated with the development of aPL, predominantly LA. Summarizing the relevant studies published between 1976 and 1998, Dlott and Roubey (109) have revealed the features suggestive or consistent with drug-induced LA in 13 of 42 reported cases. The authors highlighted the clinical presentation of thrombosis in 11 cases that could be associated with the underlying cardiac or vascular pathologies for which the patients were prescribed procainamide (109). Generation of β2GPI-dependent IgG aCL after the administration of procainamide resulted in thrombosis and, in one case, of the drug-induced APS (112). A number of studies demonstrated association between quinidine and its stereoisomer quinine and the occurrence of LA (113–115), while the occurrence of aCL was only rarely detected (116). Several mechanisms have been proposed to explain the link between antiarrhythmic drugs and aPL induction. The most plausible among these is a non-specific interaction of antiarrhythmic agents with membrane phospholipids, situated close to the sodium channels, which may trigger an initial event leading to self-reactivity (117).

Pharmacological efficiency of antipsychotic drugs is related to the toxic effects imposed by their non-specific interaction with the phospholipid bilayer, particularly with negatively charged lipids (118). Chlorpromazine (CPZ) is the agent most often implicated in cases of drug-induced aPL (109). LA, which is the main type of aPL antibodies found to be associated with CPZ, is rarely reported as the cause of thrombosis (109). Incidence of aCL alone or together with LA, following CPZ administration, was described in two separate studies (110, 119). Gharavi et al.(17) examined β2GPI-dependence of aPL, which is triggered by CPZ, and found its resemblance with autoimmune type antibodies (17). In another study, however, the presence of aCL, anti-β2GPI or LA was detected in patients with psychiatric diseases regardless of treatment by CPZ or other antipsychotics (120).

Besides antimicrobial activities, antibiotics may impose substantial interference with the host physiology and metabolism (121). Amoxicillin (111) and streptomycin (113) were the first antibiotics associated with the LA activity. In a later study, production of transient IgG aCL in response to sulfasalazine has been reported (122). There has been a renowned interest regarding the influence of antibiotics on aPL formation. Two studies reported the occurrence of LA, anti-β2GP1 and anti-phosphatidylethanolamine during the course of minocycline therapy (123, 124). The phenomenon of antibiotics-related aPL is arguable as it may be a consequence of the infectious process itself.

Other drugs, including fluvastatin, propylthiouracil, carvedilol, adalimumab, infliximab, and thalidomide and chemical agents such as acrylamide and silicone are suspected as being associated with aPL production but there is only a limited number of studies available. At least 10 cases of aPL-mediated vasculitis and/or thrombotic manifestations were attributed to cocaine abuse (125–127). It was suggested that certain cocaine-related clinical presentations can be related to the toxic effect of adulterant levamisole (antihelminth agent) or their synergic action.

The exact mechanisms of the drug-induced autoimmune reactions are not definitely known. Several pharmacological agents, including procainamide, quinidine, valproic acid, and phenytoin inevitably destroy the integrity of phospholipid bilayer by passing through the cell membrane to reach their site of action (128). Membrane perturbations may be implicated in altered presentation of neo-antigens or cryptic epitopes resulting in the consequent autoimmune responses. Among others, individual acetylation status and hapten-like characteristics of a particular drug may be considered as possible mechanisms for drug-induced aPL.

## Other Environmental Factors

Resident gut microbiota, together with other environmental factors such as nutritional status and stress, can play a crucial role in immune homeostasis (129). Numerous experiments carried out to understand whether dietary manipulations can result in a loss of self-tolerance have revealed compelling effects of daily calories, proteins or fat intake on the development of autoimmune diseases (130, 131). In particular, the diets containing omega-3-rich linseed oil as a fat source have demonstrated a reduction in the serum levels of aCL and anti-dsDNA antibodies in BALB/c mice with experimentally induced lupus (130). The fatty acid composition of cell membrane scan be influenced by the phospholipids ingested. Alterations of membrane structure and fluidity, observed in response to dietary fats, might result in modulation of functional activities of macrophages, dendritic cells, or T lymphocytes (132). Another mechanism, attributed to the immunomodulatory effects of omega-3, is the reduction of eicosanoid synthesis, which is an important mediator regulating secretion of cytokines and inflammatory gene expression (133). In autoimmune and thrombosis-prone W/BF1 mice, feeding a calorie-restricted diet resulted in the reduction of aCL and anti-dsDNA and in protection against thrombogenesis (134). Mechanisms of protective effect of calorie restriction against autoimmunity remain debatable, though it appears that abrogation of free radical generation and inflammatory responses may be involved (135).

Several studies emphasized a possible association of low serum vitamin D levels and/or high frequency of vitamin D insufficiency with hypercoagulable state in APS patients (136). Besides, vitamin D may inhibit the anti-β2GPI-stimulated TF expression in human umbilical vein endothelial cells (HUVECs) has been shown (137). Vitamin D alone as well as in combination with low molecular weight heparin (LMWH) may exert an anti-inflammatory effect on aPL-stimulated trophoblasts (138). Thus, vitamin D supplements may warrant further investigation as a promising prophylactic agent for preventing aPL-related thrombotic and obstetric complications.

## Mechanisms of aPL Production

A variety of mechanisms have been suggested to explain the origin of aPL. Production of aPL is a complex and largely unknown process, encompassing a multifactorial interaction of genetic susceptibility variants and environmental factors. Infectious agents may cause the development of aPL through antigen-dependent mechanisms such as molecular mimicry, or in antigen-independent manner such as breakdown of immune tolerance due to inflammation.

Currently molecular mimicry is considered to be as the most prevalent explanation for a frequent association of infections and aPL-related clinical manifestations and APS. Molecular mimicry represents a phenomenon of structure-and-function similarity between foreign and host antigens. As a result, the self-tolerance is compromised and the pathogen-specific immune response cross-reacts with self-antigens. There is a strong evidence for a significant homology between the bacterial and viral structures and peptides derived from β2GPI, which contribute to the selection of cross-reactive T and B cells. The molecular-mimicry hypothesis was tested in several animal model experiments designed to evaluate the pathogenic potential of infectious agents bearing membrane proteins similar to the main immunogenic epitopes targeted by anti-β2GPI antibodies. Induction of aPL in animals by heterologous β2GPI alluded that autoimmune reaction may be elicited by the immune complexes formed by host PL and PL-binding proteins derived from common infectious agents (139). Biopanning procedures identified synthetic peptides with high homology to the different regions of β2GPI, which, in *in vivo* and *in vitro* experiments, diminished endothelial cell activation, and adhesion properties of monocytes caused by patient-derived anti-β2GPI (140). This group further showed a significant rise of aCL and anti-β2GPI in mice immunized with *Haemophilus influenzae, Neisseria gonorrhoeae, Candida albicans*, or tetanus toxoid, which share structural homology with one of selected hexapeptides (TLRVYK) (141). As a confirmation to the earlier reports, APS-related clinical and serological manifestations were corroborated in naive mice passively infused with anti-TLRVYK antibodies from immunized mice (141). Similar results were obtained when mice were immunized with other synthetic peptides of bacterial or viral origin (adenovirus, CMV, *Bacillus subtilis*) that shared sequence and functional similarity with a 15 amino acid peptide “GDKV” in major phospholipid-binding region of β2GPI (139). Induction of aCL and anti-β2GPI in immunized mice supported the idea that certain viral and bacterial agents may trigger autoreactive aPL response via interaction of infection-derived PL-binding peptides with host β2GPI.

Infection(s) can cause conformational changes in β2GPI resulting in anti-β2GPI formation. Particularly, β2GPI was shown to interact with *Streptococcus pyogenes* surface protein H, thereby exposing a cryptic epitope within domain I of β2GPI and triggering aPL response (142). Additionally, alteration of host antigenic determinants due to tissue injury and generation of neoepitopes may lead to molecular mimicry. Inflammation may play a role in modification of proteins, thus providing a source of neoepitopes that can be recognized by antibodies as a non-self. In the context of innate immune activation and a pro-inflammatory environment, interaction of β2GPI with anionic PL may result in the presentation of novel T cell epitopes, which are not observed in non-autoimmune individuals (143) and which appear as a result of modulation of presentation pathways (144). Among inflammatory cascades, oxidative stress is a key mechanism, which affects post-translational modification of proteins and/or alters membrane bilayers via lipid peroxidation (145). In addition, reactive species may enhance immunogenicity of β2GPI. Elevated levels of circulating complexes, which are formed by oxidatively modified low density lipoproteins (oxLDL) and β2GPI (oxLDL/β2GPI) and recognized by anti-β2GPI, were found to be associated with the increased risk of arterial thrombosis in APS and SLE patients (146).

In addition to the infection-induced β2GPI immunogenicity, certain conformational changes may lead to the exposure of the major B-cell binding site on domain I and of the major T-cell binding site on domain V that are required for aPL response (147). Each of the five β2GPI domains has been implicated in interaction with antibodies. The high immunogenicity and pathogenicity of antibodies with reactivity against DI of β2GPI, however, is a newly recognized circumstance. It is thought that β2GPI in the serum is present predominantly in a “closed” circular conformation, in which the interaction of domain I with domain V prevents the exposure of the G39-R43 epitope. Upon the conformational change, induced by the interaction of β2GPI with anionic surfaces (“open” conformation), domain I opens up thus exposing a hidden epitope and triggering the formation of autoantibodies (148). Multiple epidemiological studies have demonstrated that antibodies against DI correlate with a substantially increased risk of thrombosis and, to a lesser extent, of pregnancy complications (149–151). This is especially relevant for the patients with a triple aPL positivity, that confers the highest risk for thrombotic events and more severe forms of APS (152–154). One of the mechanisms responsible for the increased thrombosis rates is an anti-D1-mediated increase in monocytic TF expression (our unpublished data). A very recent study challenged the pathogenic value of antibodies to domain 1 or 4/5 of β2GPI in terms of risk stratification of APS-related complications *in-vitro*. Potent procoagulant properties of anti-D1 were demonstrated in LPS-primed rats, while anti-D5, similarly to negative control, was unable to promote clot formation (155). Despite this, there is still a controversy on whether the predictive power of classic aPL tests will benefit from anti-D1 screening (154) or not (153).

Anionic compounds with the ability to bind β2GPI appeared to be an interesting potential player in the pathogenic loop that supports continuous generation of anti-β2GPI in APS (144). The model proposed suggests that the FcγRI-mediated uptake of β2GPI/anti-β2GPI/anionic surface complexes by macrophages results in recognition and presentation of the β2GPI cryptic determinants. A subsequent activation of β2GPI-reactive CD4+ T cells triggers anti-β2GPI production by B cells thus establishing a self-sustained loop (144). Under physiological conditions, systemic oxLDL, which are phospholipids expressed on activated platelets or presented on apoptotic bodies, may potentially act as a source of anionic substrates that form stable complexes with β2GPI to drive aPL response via the mechanism described above. In a focused review by Andreoli and coworkers, pathogenic interplay between aPL and apoptotic processes was emphasized (156). The authors pointed out that apoptotic cells may act as immunogenic factors capable of triggering the aPL response. On the other hand, the persistent exposure of aPL target by dying cells may promote autoimmunity through the maintenance of inflammation, especially in the settings with the increased IFN signature (156).

Notwithstanding of the numerous mechanisms proposed, it is not clear how aPL antibodies induce thrombosis and obstetric complications. It was suggested that the sustained aPL titers and procoagulatory state “first hit” precipitate thrombotic events only in the presence of inflammatory “second hit” (157). Factors such as trauma, vascular injury, surgery, pregnancy, age, hypertension, diabetes, obesity, and infection may also potentiate thrombus formation (158, 159).

The etiology of APS is attributed to the interplay between genetic and environmental insults. Nevertheless, the impact of environment on disease risk is difficult to evaluate and the mechanisms involved remain pressing and enigmatic. Evidences accumulated over decades indicate that gene-environment interactions, mediated at the epigenetic level, may trigger autoimmune processes (160). Epigenetic alterations may regulate CD4, CD8 and B cell functions and appears to drive maturation of the high-affinity antibody response (161, 162). It is therefore intriguing to investigate the influence of particular environment factors on epigenetic landscape and following aPL response.

Our review includes the wide range of publications describing environmentally-triggered aPL generation including research papers, case reports, case-control studies, reviews, and clinical, epidemiological, population-based and big-cohort studies. Despite this, we are aware that the data in this review might be limited for several reasons. The first is the biased/heterogeneous or limited data of the relevant reviewed studies. The second limitation concerns the laboratory procedures for aPL detection, which still lack of standardization and interpretation strategies and may vary among laboratories. The possible variation arising from non-uniformity of results interpretation were not considered here.

## Conclusion

**Figure 1:**
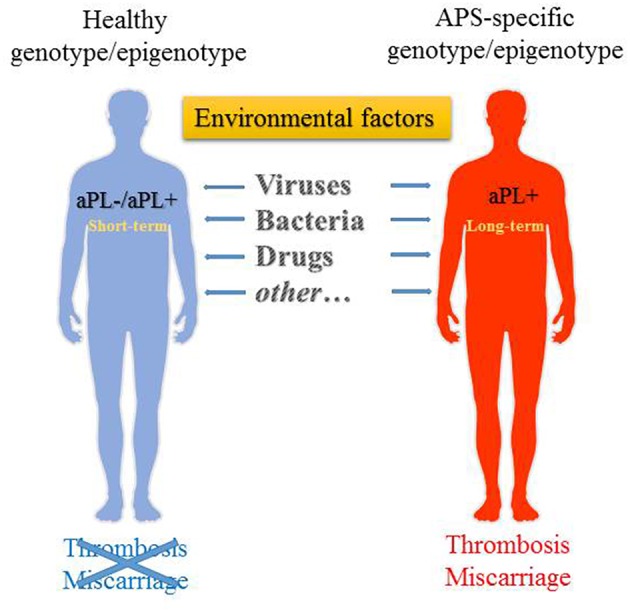
Environmental factors that may trigger the generation of aPL. aPL generation can be triggered in both genetically susceptible as well as non-susceptible individuals, albeit it is transient in the latter. Long-term persistence of aPL in predisposed individuals may then lead to the development of APS.

In summary, the environmental triggers such as bacteria, viruses, commensal bacteria, vaccines, drugs, and other factors are potentially capable of inducing a variety of aPL and causing APS through a wide range of mechanisms in genetically susceptible individuals (Figure 1). These factors are also specified in Table 1. A most compelling evidence supporting the role of environmental factors in induction of aPL is largely obtained from the epidemiological studies. The presence of aPL in healthy individuals points to the normal physiological role played by aPL. The post-infection increase of aPL is usually transient, and it is not accompanied by the manifestations of APS such as thrombosis. The main question remains, however, why in a genetically susceptible host the increase of aPL in response to the environmental stimuli is not resolved and results in the transition from low-affinity to high-affinity autoantibodies, with the increased pathogenic potential of aPL. Presently our understanding of mechanisms of inflammation resolution and self-tolerance is rather limited and cannot explain why in a subset of population initial adaptive immune response against foreign antigens is rerouted against self-antigens. Does it involve compromised innate immunity mechanisms ensuing continuous activation via the DAMP signaling? Or is adaptive immune response in the later stages involved that prevents clonal selection of T and B cells producing self-antibodies? Uncovering the mechanisms behind the generation of heterogeneous aPL that contribute to the development of APS will help to design the strategies for treatment and prevention of this disease.

**Table 1:** Bacterial, viral, and parasitic agents linked to the aPL production and clinical manifestation.

	**aCL**	**aβ2GPI**	**LA**	**Thrombosis**	**Structure**	**References**
**VIRAL INFECTIONS**						
HIV	+	+	+	+	Enveloped	(12–15)
Hepatitis C	+	+	+	+	Enveloped	(13, 15, 21)
Parvovirus B19	+	+	+	+	Non-enveloped	(23–25, 63)
CMV	+	+	+	+	Enveloped	(31, 63)
Varicella zoster virus	+	+	+	+	Enveloped	(34–37, 63)
Epstein-Barr virus (EBV)	+	+	+	+	Enveloped	(13, 38, 39, 63)
Adenovirus	+	+	−	−	Non-enveloped	(15, 15, 63)
Hepatitis A	+	−	−	+	Non-enveloped	(63)
Influenza	−	−	−	+	Enveloped	(63)
Hepatitis B	+	−	−	−	Enveloped	(22, 63, 101)
Rubella	+	−	−	−	Enveloped	(28, 63)
Measles	−	−	−	−	−	(28)
Hepatitis D	+	+	+	−	Enveloped	(22)
Human T-lymphotropic virus 1	+	−	−	−	Enveloped	(12, 63)
Mumps	+	−	−	−	Enveloped	(28, 63)
**BACTERIAL INFECTIONS**						
*Coxiella burnetii*	+	−	+	−	Gram-negative	(28, 63)
*Mycoplasma pneumonia*	+	+	−	+	Gram-negative	(42, 53, 55–57, 63)
*Streptococcus* spp.	+	+	+	+	Gram-positive	(28, 52, 63)
*Mycobacterium tuberculosis*	+	+	−	+	Not classified	(28, 63)
*Mycobacterium leprae*	+	+	+	+	Gram-positive	(62–66)
*Borrelia burgdorferi*	+	+	+	+	Gram-positive	(125)
*Staphylococcus* spp.					Gram-negative	(28, 52, 58, 59)
*Treponema pallidum*	+	+	−	−	Gram-negative	(10, 13, 63)
*Escherichia coli*	+	+	−	+	Gram-negative	(28, 52, 63)
*Salmonella* spp.	+	+	+	+	Gram-negative	(28, 63)
*Klebsiella* spp.	+				Gram-negative	(10, 28)
*Fusobacterium necrophorum*	+	−	+	+	Gram-negative	(60, 63)
*Chlamydiae*	+	+	−	−	Gram-negative	(28, 63)
*Helicobacter pylori*	+	+	−	−	Gram-negative	(75, 76)
**PARASITIC INFECTIONS**						
*Plasmodium malariae*	+	+	−	+	Intracellular parasites	(12, 13, 63)
*Plasmodium falciparum*	+	−	−		Intracellular parasites	(68)
*Leptospira* spp.	+	+	−	−	Extracellular parasites	(63)

## Author Contributions

All authors listed have made a substantial, direct and intellectual contribution to the work, and approved it for publication.

### Conflict of Interest Statement

The authors declare that the research was conducted in the absence of any commercial or financial relationships that could be construed as a potential conflict of interest.
